# 2D speckle tracking echocardiography and comparison with cardiac magnetic resonance in children with acute myocarditis

**DOI:** 10.3389/fcvm.2024.1446602

**Published:** 2024-10-25

**Authors:** M. Burešová, J. Pavlíček, P. Hanzlíková, H. Tomášková, O. Rybníček

**Affiliations:** ^1^ Department of Pediatrics, University Hospital Ostrava, Ostrava, Czechia; ^2^ Faculty of Medicine, University of Ostrava, Ostrava, Czechia; ^3^ Faculty of Medicine, Masaryk University, Brno, Czechia; ^4^ Biomedical Research Center, FN Hradec Králové, Hradec Králové, Czechia; ^5^ Department of Imaging Methods, University Hospital Ostrava, Ostrava, Czechia; ^6^ Department of Epidemiology and Public Health, Faculty of Medicine, University of Ostrava, Ostrava, Czechia; ^7^ Department of Pediatrics, University Hospital Brno, Brno, Czechia

**Keywords:** myocarditis, PIMS-TS, COVID-19, speckle tracking echocardiography, global longitudinal strain of the left ventricle, cardiac magnetic resonance

## Abstract

**Background:**

Cardiac magnetic resonance (CMR) plays a major diagnostic role in acute myocarditis (AM) in children as biopsy is rarely performed in this age group. Contribution of standard echocardiography (ECHO) is limited in AM, but speckle tracking echocardiography (STE) quantitatively characterizes myocardial function, with good sensitivity for detecting subclinical left ventricular (LV) dysfunction and regional kinetics disorders beyond the site of inflammation. This work aimed to evaluate the diagnostic potential of STE as compared with CMR findings in pediatric patients with AM.

**Methods:**

The study was conducted during 2022–2023. Troponin, electrocardiography, ECHO with STE, and CMR with early and late enhancement were performed on each patient. Affected heart segments were analyzed by both STE and CMR, and the correlation of the two methods was assessed.

**Results:**

During the study period, 20 children were diagnosed with AM [14 boys, 6 girls; mean age 12 years (median 14)]. On ECHO, three patients had a deviation in LV biometry, and four patients had a mild systolic function disorder. STE showed at least one affected cardiac segment in all patients, most often the inferolateral segment (16/20; 80%). Of the 20 patients, STE showed a reduction in LV global longitudinal strain in 13 (65%) patients. In all patients, CMR identified an inflammatory focus, most frequently inferolateral (15/20; 75%). The strongest accordance between STE and CMR was observed for the involvement of anterolateral segments (k = 0.88) and the weakest for inferoseptal damage (k = 0.4).

**Conclusions:**

STE can provide important diagnostic information in pediatric patients with AM. This modality supports the detection of early regional edema and subclinical myocardial dysfunction and can determine the impairment severity. STE is non-invasive and repeatable without the need for special patient preparation or for general anesthesia.

## 1 Introduction

Myocarditis is an inflammatory disease of the heart muscle, defined by histological or immunohistochemical findings or findings on magnetic resonance imaging. The etiology can be infectious or non-infectious (1, 2). Clinically, a fulminant, acute, or chronic form of myocarditis can occur, with symptoms ranging from non-specific manifestations to heart failure, cardiogenic shock, or sudden death.

The diagnosis of myocarditis is based on a comprehensive evaluation of the patient's history, clinical symptoms, laboratory parameters (troponin elevation is the key finding), electrocardiography, and imaging techniques. A significant advancement in the diagnosis of myocarditis came with improvements in the technical parameters and interpretation of cardiac magnetic resonance (CMR) findings, allowing confirmation of myocarditis without an endomyocardial biopsy (EMB) (1), which has been considered the reference standard for diagnosis.

Among possible myocarditis-specific imaging methods, standard echocardiography (ECHO), as the standard method for morphological description and assessment of systolic and diastolic function, is usually performed first in a child with suspected myocarditis. When measuring myocardial kinetics, however, ECHO is significantly limited in diagnostic accuracy compared with quantitative measurement of regional myocardial deformation using two-dimensional speckle tracking echocardiography (STE). STE is non-invasive and quantitatively characterizes myocardial function by evaluating myocardium deformation, yielding better sensitivity for detecting subclinical left ventricular (LV) dysfunction (3). What is not clear is whether STE can facilitate or clarify a diagnosis of myocarditis in childhood, or even replace CMR if the latter is not available or cannot be performed.

This work aimed to evaluate and identify regional LV myocardial kinetics disorders using two-dimensional STE and compare the findings with those from CMR in pediatric patients with acute myocarditis.

## 2 Methods

### 2.1 Design

This study was conducted from January 2022 to December 2023 at the Department of Pediatrics, University Hospital Ostrava, Czech Republic. This tertiary referral center for pediatric cardiology serves a population of about 1,200,000 inhabitants, with 11,000 live births per year. The study included patients with a diagnosis of acute myocarditis confirmed according to all of the following: the clinical course, laboratory findings, and CMR. Troponin, ECG, ECHO, and CMR examinations were performed in each patient. The study was approved by the ethics committee of the University Hospital Ostrava under reference number 147/2024. There was no patient or public involvement in this research.

### 2.2 Echocardiography

The GE Vivid E95 (GE Healthcare, Horten, Norway) with advanced special software allowing speckle tracking for LV chamber evaluation (automated function imaging) was used for echocardiography. Advanced tools in the EchoPAC PC workstation equipped with the 2D strain research package were used for speckle tracking assessment for both ventricles.

For basic LV measurement, we used the technique according to Teichholz. The main parameters included the dimensions of the interventricular septal (IVS) and the LV posterior wall (LVPW) diameters at the end diastole, and the LV internal diameter at the end diastole and end systole. We converted the results into Z-scores using the official calculator for the Czech Republic, designed by the Prague Heart Center (4). Systolic function was evaluated by calculating the LV ejection fraction (EF) and fractional shortening (FS) according to Teichholz.

The 2D speckle tracking was performed at a rate of 60–80 frames/s from three viewing planes (apical long axis, apical four-chamber, and two-chamber planes) and evaluated using a semi-automatic algorithm (EchoPac, GE Healthcare). Aortic valve occlusion was measured on the 2D image. The region of interest was adjusted to cover only the thickness of the myocardium (5). The LV was then divided into 17 segments covering the entire LV myocardium, and global longitudinal strain (GLS) was calculated automatically as the mean global peak systolic strain from each of the three imaging planes.

### 2.3 CMR

During the first week of hospitalization, CMR was performed on a Siemens 3 T Magnetom Prisma, SW XA 31 device or a Siemens 1.5 T Magnetom Sola, SW XA 51 device. The so-called steady-state free precession sequence constitutes a basic part of the CMR protocol, giving high spatial resolution and providing a precise image for assessment of myocardial kinetics. Visualizing the different proton densities of individual tissues was crucial for distinguishing tissue CMR characteristics. The myocardium was evaluated on T1- and T2-weighted images, and all patients received a gadolinium-based contrast agent with immediate and delayed imaging. Gadovist 1 mmol/ml (dose 0.1 ml per kg; Bayer Pharma AG, Germany) was used as a contrast agent in children over age 7 years. For children under age 7 years, Dotarem 0.5 mmol/ml (dose 0.2 ml per kg; Guerbet, France) was used. The updated Lake Louise criteria were used to assess for the presence of myocarditis (6, 7).

The examinations were carried out according to a standard protocol. First, a morphological-kinetic examination of the heart was performed in basic projections (four-chamber, two-chamber for both ventricles, followed by the evaluation of the entire volume of the heart in the short axis). For these images, coherent echo (steady-state free precession) trueFISP sequences were used with reverse data segmentation according to synchronization with the ECG recording. CMR parameters were selected as follows: image width 6 mm, matrix 192 × 192, acquisition time 9 s, reverse segmentation in 25 phases in 1 R-R interval. This was followed by a perfusion examination of the myocardium with intravenous administration of a contrast agent. The turboFLASH dynamic sequence was initiated at the moment of the contrast agent application. After 10 min from the administration of the contrast agent, the inversion recovery FLASH sequences were performed, supplemented in selected parts of the myocardium suspected to be impaired by the “phase-sensitive inversion recovery” sequence—PSIR (image width 6 mm).

In three patients, the examination was performed under general anesthesia; in another five, light sedation was applied.

### 2.4 Statistical analysis

To describe the data, we applied basic descriptive statistics (arithmetic mean, standard deviation, median, interquartile range). Skewness and kurtosis tests of normality were applied. A symmetry test was used to compare the results of the CMR and ECHO methods, and agreement was assessed with the kappa index (8), expressed in terms of positive and negative results and overall agreement. Statistical test results were evaluated at a significance level of 5%, and all analyses were conducted using Stata version 17.

## 3 Results

### 3.1 Basic evaluation

During the 2022–2023 study period, 20 children were hospitalized with a diagnosis of acute myocarditis. Of the total number, 70% (14/20) were boys and 30% (6/20) were girls. The mean age was 12.0 years (median 14.0 years; interquartile range 9–16.8 years; min–max 2–18 years). The mean length of hospitalization was 15 days (median 14 days; interquartile range 11–17 days; min–max 8–26 days). From the clinical manifestations, non-specific symptoms similar to a viral disease dominated in 70% (14/20) of patients, and 30% (6/20) reported chest pain. ECG abnormalities (ST segment changes) were identified in 25% (5/20) of patients. The initial levels of troponin and N-terminal pro-B-type natriuretic peptide are shown in Table 1. In 85% (17/20) of the patients, a possible etiology for myocarditis was identified. In 65% (13/20), the myocardial involvement met the diagnostic criteria for the multisystem inflammatory response syndrome associated with SARS-CoV-2 (i.e., pediatric inflammatory multisystem syndrome temporally associated with SARS-CoV-2; PIMS-TS), and in 20% (4/20), we identified specific IgA and IgM antibodies for Coxsackie B1, B2, and B3 and enteroviruses. PCR confirmed the presence of influenza A in one patient; in two cases, no possible agent was identified. EMB was not indicated in any case.

**Table 1:** Laboratory results and echocardiographic measurements.

	Mean	Median	IQR	Min	Max
	Laboratory				
Troponin I (ng/L)	4,232	495	230–3,630	79	42,221
NT-proBNP (pg/ml)	13,125	3,263	284–21,673	77	57,387
	Echocardiography: biometry and systolic function				
IVS (Z-score)	0.651	0.29	−0.1–1.4	−0.69	3
LVID (Z-score)	0.356	0.42	−0.22–0.98	−2.3	3.2
LVPW (Z-score)	0.857	0.85	0.2–1.4	−0.66	3.1
LVEF (%)	63	63	61–67	48	77
LVFS (%)	31	33	27–36	22	43
	Global longitudinal strain^a^				
GLS LV	−16.6	−16	−18 to −15.4	−21.3	−13

LVEF, left ventricular ejection fraction; LVFS, left ventricle fractional shortening; GLS, global longitudinal strain; IVS, interventricular septum; LV, left ventricular; LVID, left ventricular internal diameter; LVPW, left ventricular posterior wall; NT-proBNP, N-terminal pro b-type natriuretic peptide.

^a^ GLS is given in negative values; towards 0 indicates pathology, GLS < −16% abnormal, GLS > −18% normal, GLS −16% to −18% = grey zone.

### 3.2 ECHO

During ECHO, overall cardiac morphology, LV biometry, and systolic function were evaluated. Congenital heart defect nor another morphological pathology was found in none of the patients. When measuring the walls of the left ventricle, according to the Z-score, three patients with a deviation of >2 were identified (in one patient, this deviation was solely in the IVS parameter; in two patients, it was observed in both IVS and LVPW); normal LV biometry was found in all remaining patients. Mildly reduced systolic function (48%–55%) was detected in four patients, while no significant diasystolic dysfunction was identified in any patient. No overtly abnormal wall motion or LV geometry abnormality was evident on ECHO in any patient. Four patients had Degree I–II mitral regurgitation according to a semiquantitative assessment by color Doppler mapping. No pericardial effusion was present in any patient. Selected cardiac function parameters are shown in Table 1.

### 3.3 Speckle tracking and magnetic resonance

Four segments most commonly affected by myocarditis (inferolateral, anterolateral, inferoseptal, and anteroseptal segments), i.e., with the greatest regional reduction in longitudinal stress, were used for the comparison between CMR and STE. In all patients, regional reduction in at least one of these segments was observed: inferolateral (80%; 16/20 patients), anterolateral (65%; 13/20), inferoseptal (40%; 8/20), and anteroseptal (30%; 6/20). The global longitudinal tension of the left ventricle was reduced in 65% (13/20) of patients.

CMR confirmed the finding of acute myocarditis in the LV myocardium in all patients, while right ventricular involvement was not found in any patient. Of the segments selected for comparison (see above), evidence of inflammatory involvement was detected in at least one of these segments in all patients, namely: inferolateral (75%; 15/20), anterolateral (70%; 14/20), inferoseptal (50%; 10/20), and anteroseptal (40%; 8/20).

**Figure 1:**
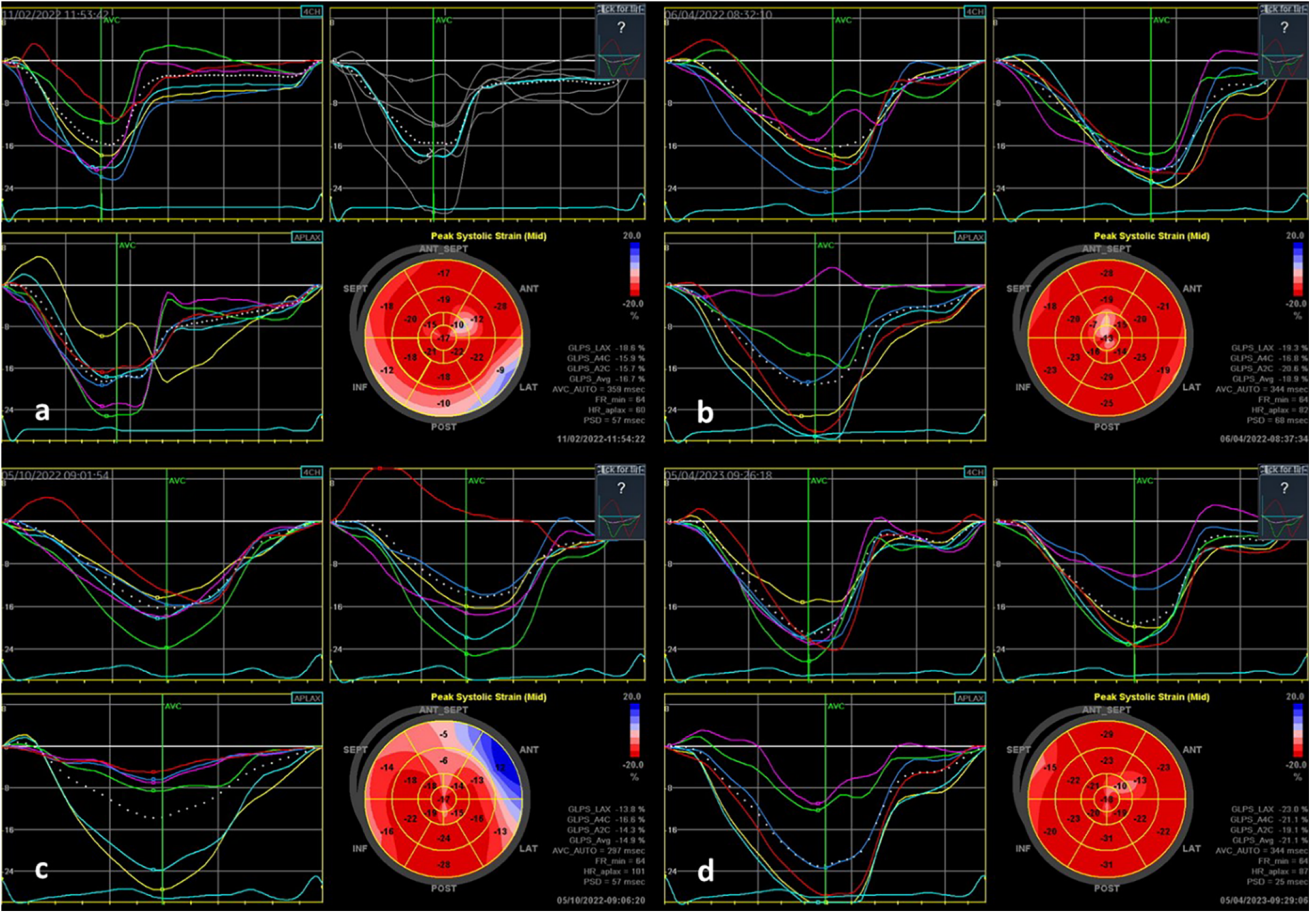
STE findings in two patients with acute myocarditis: **(a)** a 9-year-old PIMS-TS patient with transient mild LV dysfunction—longitudinal strain bull's eye plot patterns showing the involvement in the basal inferolateral to inferior and the mid-anterolateral area; **(b)** follow-up examination of this patient after 3 months, showing normalization of findings in the affected area; **(c)** an 11-year-old patient with PIMS-TS with preserved good LV function—longitudinal strain bull's eye plot showing the involvement in the anterolateral area and in the septal direction; **(d)** follow-up examination after three months, showing normalization of findings in the affected area.

**Figure 2:**
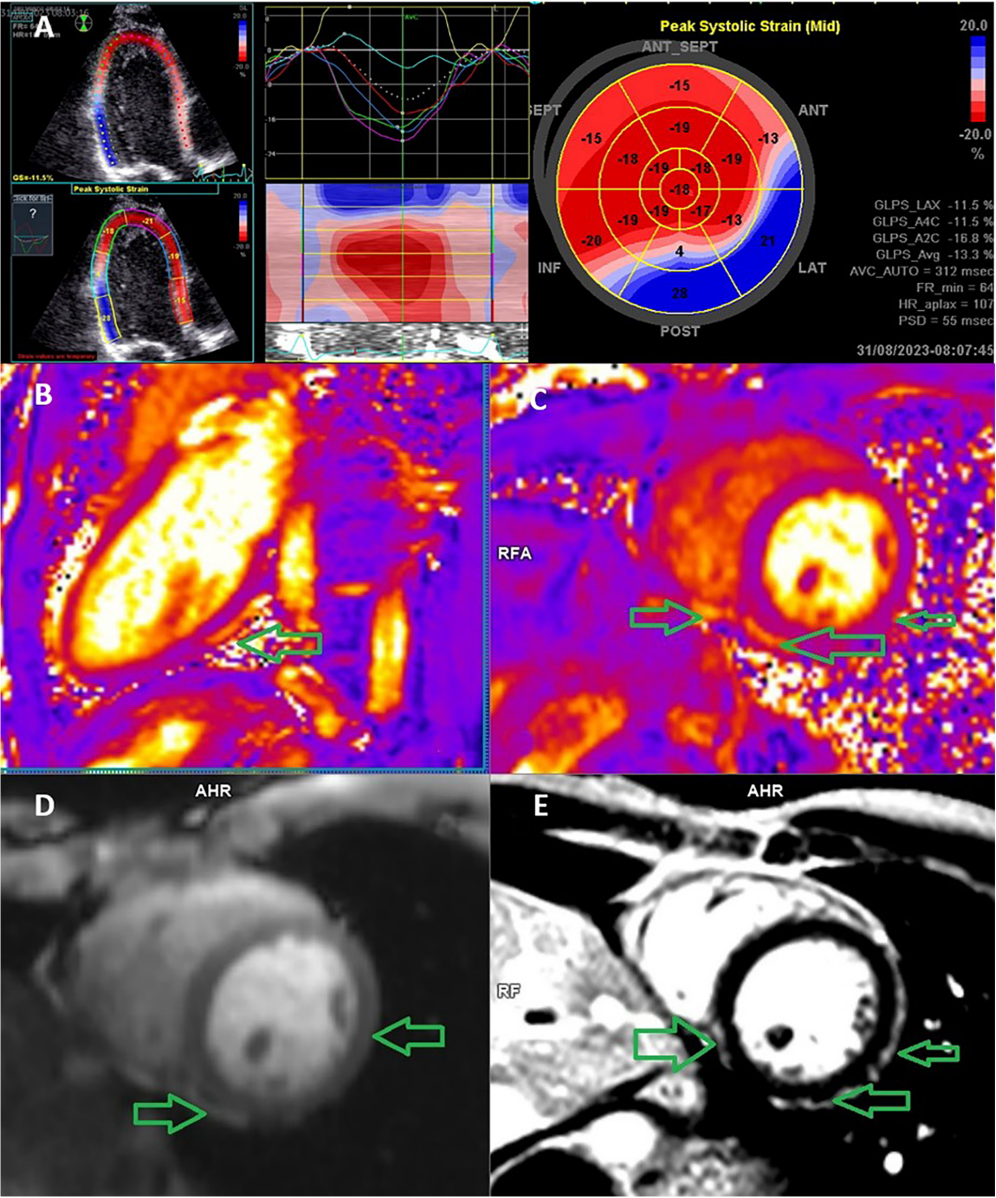
Echocardiography speckle-tracking strain analysis and cardiac MRI in a 17-year-old man with acute perimyocarditis. **(A)** Speckle-tracking assessment of segmental and global strain on an apical long-axis echocardiography view with a bulls-eye plot of regional longitudinal strain values, which demonstrates impaired strain at the basal inferior and inferolateral wall. The blue segment is dyskinetic, meaning that the wall segment is bulging when it should be contracting. If the left ventricle walls are dyskinetic (i.e., bulging out), the GLS value is positive. **(B)** Cardiac MRI image demonstrates a T2 relaxometry native dual-chamber scan; the arrow indicates the area of the perimyocardial edema. **(C)** T2 relaxometry native short axis scan; the arrow indicates the area of the perimyocardial edema. **(D)** T1 DCE (dynamic contrast enhancement)—early enhancement of the inflammation in the peri-myocardial position of the chamber wall showing an enhancement of the near pericardial layer typical of myocarditis. **(E)** T1 PSIR postcontrast 15 min, short-axis scan, the arrow indicates the area of peri-myocardial scarring. The left ventricular ejection fraction (global function) was reduced. MRI: magnetic resonance imaging.

A comparison of the methods revealed that CMR and STE agreed on the localization in the only patient with only one segment affected. In all other patients, more than one segment was affected. In all these patients, STE and CMR agreed on at least one affected segment. In effect, we can say that in all patients with CMR-confirmed myocarditis, STE detected myocarditis as well. STE and CMR showed the strongest agreement in detecting the involvement of anterolateral segments (k = 0.88) and the weakest correlation in detecting inferoseptal damage (k = 0.4). The overall evaluation of agreement between STE and CMR for all four segments is shown in Table 2, and the pathological results acquired using both methods are shown in Figure 1 (pathological STE imagery from two patients), and Figure 2 (comparison of STE and CMR in one patient).

**Table 2:** Myocardial alteration in individual segments: agreement between STE and CMR.

Segment	Agreement			Test of symmetry	Kappa index	
	Positive	Negative	Total	*P*	Value	Interpretation
Inferoseptal	6 (30%)	8 (40%)	14 (70%)	0.414	0.40	Fair
Inferolateral	14 (70%)	3 (15%)	17 (85%)	0.564	0.57	Moderate
Anterolateral	13 (65%)	6 (30%)	19 (95%)	0.317	0.88	Almost perfect
Anteroseptal	4 (20%)	10 (50%)	14 (70%)	0.348	0.41	Moderate

CMR, cardiac magnetic resonance; STE, speckle tracking echocardiography.

### 3.4 Follow-up

All patients were offered re-examination of STE and CMR at 3 months; in all, 80% (16/20) of patients came for the examination. The follow-up CMR showed scarring in the affected areas, but no myocardial kinetics disorder. Echocardiographically, all patients had normal LV EF on follow-up examination (two patients at the lower limit of normal). In all but one, LV GLS fully normalized by the time of the follow-up examination. In one patient, the examination was postponed due to another illness by five months (i.e., the measurement was performed at 8 months) with normal results. In another patient, the follow-up CMR was performed 1 month after discharge due to initially impaired left ventricular function. This patient also had visible scarring; however, the kinetics was physiological and GLS LV improved significantly. Two patients did not show up for re-examination despite being repeatedly asked to come for the follow-up.

## 4 Discussion

In our region, as monitored from the position of a tertiary center, the occurrence of myocarditis in children is 0.5 per 100,000. The incidence of inflammatory heart disease has, however, been changed by the COVID-19 pandemic. We observed an increase in hospitalized myocarditis cases from 1.5 cases per 1,000 hospitalizations before the pandemic to 5 cases per 1,000 hospitalizations during the pandemic period. In terms of etiology, we originally confirmed viral diseases, but during the pandemic, inflammatory involvement of the heart predominantly occurred as a part of PIMS-TS (9, 10). Despite the change in etiology, the higher prevalence in males persisted (11).

Determination of troponin level is fundamental the diagnosis of myocarditis; today, it is largely done using hyper/ultra-sensitive methods. In myocarditis, higher troponin levels correlate with disease severity (12). A higher troponin level may reflect the extent of inflammatory involvement, but its significance along with the development of myocardial dysfunction and a subsequent adverse prognosis is not entirely clear (13). If troponin is negative, myocarditis can be excluded with a high probability (14). In our cohort, we noted a wide variation in troponin values, from only slightly increased values to those exceeding 40,000 ng/L, and all patients survived without permanent health consequences. Importantly, it needs to be pointed out that no correlation between troponin levels and systolic function disorder was observed.

In a pediatric patient with suspected myocarditis, echocardiography is always indicated. Its role is to rule out effusions and structural damage to the heart and to characterize the systolic and diastolic function of the heart and coronary arteries. Thickening of the myocardial wall can mark edema, and echocardiography can contribute to an estimation of a fulminant course (15). In this work, a slightly lower LVEF was detected in four patients; in all of them, however, LVEF normalized within a few days.

As biopsy is performed exceptionally in children and CMR has its limitations, definitive identification of damage to the heart muscle in and after acute myocarditis poses a possible problem. We, therefore, extended ECHO to include the STE modality in patients with acute myocarditis and evaluated the benefit of this method. During an examination, STE can be used to assess and quantify all basic components of myocardial function, i.e., systolic and diastolic deformation, and the rate of deformation in all three dimensions (longitudinal, circumferential, radial). At the same time, STE allows the assessment of rotation and torsion of the left ventricle and temporal relationships between individual functional components, thus offering comprehensive information on myocardial function (16). GLS is of greatest importance in STE, because compared with EF, this longitudinal systolic deformation or shortening is a more reproducible measure of LV function (when measured according to Teichholz), regardless of echocardiographic experience (17). We confirm the good feasibility and reproducibility of this examination. A longitudinal shortening alteration in at least one segment as well as heart muscle damage was confirmed in all patients. The follow-up examination, however, confirmed the early normalization of GLS and regional longitudinal strain values, which indicated good reversibility of the disability in most patients. In one patient, LV GLS remained significantly reduced even at long-term follow-up as a result of non-adherence to recommendations during recovery and early loading of the myocardium by intense sports activity.

We assessed the advantages and limitations of both methods. CMR currently dominates in the diagnosis of acute myocarditis in children (7). With the help of early gadolinium enhancement (EGE) and late gadolinium enhancement (LGE), CMR can identify reversible (intracellular edema, hyperemia, capillary leakage) and irreversible (fibrosis) damage to the myocardium. We conducted CMR in all of our patients and confirmed the acute myocarditis focus in each case, with the inferolateral segment most frequently affected. Limitations of CMR, on the other hand, may lie in the assessment of myocardial edema in EGE because of the susceptibility of T2-weighted imaging to artifacts of various etiologies, which might have reduced the reliability of the examination result. Besides, in LGE, the foci may disappear during scar retraction or fall below the limit of detectability.

In several of the younger patients in our study, it was not possible to perform parametric mapping that could be advantageous for the diagnosis of edema, fibrosis, or infiltration of the heart muscle. This was mostly due to the higher heart rates in these patients (above 90 /min); at such a high heart rate, the quality of the images deteriorates and, at our workplace, we do not bradycardize children during the examination. In some patients, another reason lied in the fact that they were under general anesthesia, precluding respiratory cooperation. It should be also mentioned that a fundamental problem with CMR lies in its availability, which can be regionally limited, and in that the assessment of findings of focal myocarditis requires considerable experience, especially considering the small heart size in children. EGE and LGE require the administration of a contrast agent, which can be contraindicated in some situations (e.g., in patients with renal impairment); moreover, in smaller children, general anesthesia may be necessary to perform CMR (18).

It is necessary to add that CMR can also measure myocardial strains using modalities such as myocardial tagging (TAG), feature tracking (FT), or fast-strain-encoded CMR imaging (fast-SENC). TAG is the oldest of those three methods, but it suffers from low spatial resolution, longer acquisition times, longer post-processing times, and the possible presence of artifacts. The advantage of FT lies in the fact that no additional acquisition is needed as it relies only on the post-processing of standard MR imagery. Thus, it comes with a possibility of retrospective evaluation from already performed standard protocols, making it suitable also for retrospective studies. The SENC method was originally limited by the necessity of holding the breath for a long time and obtaining multiple heartbeats; it has been, however, significantly improved to allow real-time acquisition of myocardial strains in a single heartbeat (fast-SENC). This method is also able to provide a description of ventricular dysfunction. All three methods provide excellent global strain reproducibility; however, TAG and SENC provide better reproducibility than feature tracking for segmental strain (19). Fast-SENC appears to be highly reproducible and suitable for serial studies and able to reliably detect subclinical changes in myocardial function (20). Fast-SENC provides an excellent description of GLS (although TAG better describes the circumferential strain) (19) and, given that GLS is the most frequently studied strain, it can be suitable as a modality of future studies. In summary, CMR strain measurements are very promising, but standardization of available techniques is necessary. On the other hand, although MR strain modalities are very promising, it is still necessary to take the practical clinical considerations into account, such as the availability and capacity of CMR, which may be a limiting factor in many hospitals. From the pediatricians’ point of view, the limitations of CMR modalities associated with high heart rates in children and the need to bradycardize the patient must be also considered.

STE is a non-invasive examination that is repeatable and usable in everyday practice. No special preparation is required and the experience of the investigator is the only limitation. The quality of the examination depends on the cooperation of the patient; age is an important factor, with older patients cooperating better. From the perspective of echocardiographic methodology, it is necessary to correctly select the appropriate probe, to ensure that the echo loops are performed correctly, and to perform simultaneous ECG.

A significant reduction in GLS and segmental involvement can be demonstrated even in asymptomatic children or in symptomatic patients with normal LV function as measured by standard techniques (21, 22). The usefulness of STE has been confirmed in acute myocarditis in both adult and pediatric studies (23–25). STE supports the detection of even small changes in the affected tissue, such as minor edema and the reaction of the surrounding tissue to it. We see the potential of STE in early detection of regional edema and associated regional subclinical dysfunction. Moreover, STE can provide additional information about the location and degree of cardiac involvement and has potential in the long-term follow-up of children after acute myocarditis. In the long term, focal myocarditis can lead to subclinical LV dysfunction and further develop into dilated cardiomyopathy. STE could be used to monitor for such subclinical dysfunction in patients after acute myocarditis to facilitate early detection of systolic failure.

In this work, we used only GLS measurements, which appear to be a stronger predictor of adverse outcomes than circumferential and radial strains because GLS reflects the function of longitudinally oriented subendocardial muscle fibers that are adversely affected in the early stages of the disease. Reference values for circumferential and radial strains have not yet been reliably established or should be used with caution.

In this study, STE and CMR were not always strongly positively correlated. Both examinations were performed in all children. Their agreement was highest in the most frequently affected (inferolateral, anterolateral) segments and lowest in the septal segments (18). In several patients, the numbers of affected segments differed between the two methods. The inferolateral and anterolateral segments were predominantly affected. In some cases, STE revealed more affected segments than CMR, which can be explained by better detection capabilities of STE (as CMR, due to the above-mentioned limitations, may sometimes fail to reveal edema). Most importantly, however, STE was capable of identifying myocarditis at least in one segment in all affected children, which suggests that if our results are confirmed on larger patient groups, it could be safely used as a substitution for CMR in diagnosing myocarditis. In view of the often limited availability of CMR, this could have a major impact on early diagnosis of myocarditis in children.

Following up on this pilot study, we plan on further elaboration of this issue and expansion of the patient group as well as including patients in whom myocarditis was not confirmed by CMR.

## 5 Conclusion

Analysis of the cardiac function using 2D STE provides important diagnostic information in pediatric patients with acute myocarditis. In contrast to standard echocardiographic methods, this modality offers the possibility of early detection of subclinical myocardial dysfunction. It can provide additional information about the severity of cardiac involvement and reliable information about the localization of myocardial involvement at the segmental level. STE is non-invasive, does not require anesthesia or the administration of a contrast agent, and is repeatable without special preparation of the patient. It is very gentle for the pediatric population and could replace CMR in indicated cases. Another significant advantage could lie in long-term follow-up monitoring of patients who have experienced cardiac inflammation, especially of patients with permanently reduced LV GLS and preserved LVEF from the perspective of monitoring for the potential development of heart failure in adulthood.

## Data Availability

The raw data supporting the conclusions of this article will be made available by the authors, without undue reservation.

## References

[1] Law YM Lal AK Chen S Ciháková D Cooper LT Jr Deshpande S Diagnosis and management of myocarditis in children: a scientific statement from the American Heart Association. Circulation. (2021) 144(6):e123–35. 10.1161/CIR.0000000000001001 34229446

[2] Bracamonte-Baran W Čiháková D. Advances in experimental medicine and biology. Adv Exp Med Biol. (2017) 1003:187–221. 10.1007/978-3-319-57613-8_10 28667560 PMC5706653

[3] Naik RJ Kiene A Patel J. Utility of 2D-speckle tracking echocardiographic strain to predict late gadolinium enhancement on MRI in children with myocarditis. J Am Coll Cardiol. (2021) 77(Supplement 1):508. 10.1016/S0735-1097(21)01867-2

[4] Kovanda J. Calculator for Pediatric Echocardiography. Available online at: http://echocalc.aspfree.cz/

[5] Voigt JU Pedrizzetti G Lysyansky P Marwick TH Houle H Baumann R Definitions for a common standard for 2D speckle tracking echocardiography: consensus document of the EACVI/ASE/industry task force to standardize deformation imaging. Eur Heart J Cardiovasc Imaging. (2015) 16(1):1–11. 10.1093/ehjci/jeu184 25525063

[6] Joudar I Aichouni N Nasri S Kamaoui I Skiker I. Diagnostic criteria for myocarditis on cardiac magnetic resonance imaging: an educational review. Ann Med Surg (Lond). (2023) 85(8):3960–4. 10.1097/MS9.0000000000001040 37554854 PMC10406012

[7] Ferreira VM Schulz-Menger J Holmvang G Kramer CM Carbone I Sechtem U Cardiovascular magnetic resonance in nonischemic myocardial inflammation: expert recommendations. J Am Coll Cardiol. (2018) 72(24):3158–76. 10.1016/j.jacc.2018.09.072 30545455

[8] McHugh ML. Interrater reliability: the kappa statistic. Biochem Med (Zagreb). (2012) 22(3):276–82. 10.11613/BM.2012.031 23092060 PMC3900052

[9] Musilová T Jonáš J Gombala T David J Fencl F Klabusayová E COVID-19-associated paediatric inflammatory multisystem syndrome (PIMS-TS) in intensive care: a retrospective cohort trial (PIMS-TS INT). Children (Basel). (2023) 10(2):348. 10.3390/children10020348 36832477 PMC9955007

[10] David J Stara V Hradsky O Tuckova J Slaba K Jabandziev P Nationwide observational study of paediatric inflammatory multisystem syndrome temporally associated with SARS-CoV-2 (PIMS-TS) in the Czech Republic. Eur J Pediatr. (2022) 181(10):3663–72. 10.1007/s00431-022-04593-7 35987943 PMC9392434

[11] Fairweather D Beetler DJ Musigk N Heidecker B Lyle MA Cooper LT Jr Sex and gender differences in myocarditis and dilated cardiomyopathy: an update. Front Cardiovasc Med. (2023) 10:1129348. 10.3389/fcvm.2023.1129348 36937911 PMC10017519

[12] Chong D Chua YT Chong SL Ong GY. What raises troponins in the paediatric population? Pediatr Cardiol. (2018) 39(8):1530–4. 10.1007/s00246-018-1925-5 29923133

[13] Kobayashi D Aggarwal S Kheiwa A Shah N. Myopericarditis in children: elevated troponin I level does not predict outcome. Pediatr Cardiol. (2012) 33(7):1040–5. 10.1007/s00246-012-0222-y 22322566

[14] Guyther J Cantwell L. Big tests in little people. Emerg Med Clin North Am. (2021) 39(3):467–78. 10.1016/j.emc.2021.04.003 34215397

[15] Felker GM Boehmer JP Hruban RH Hutchins GM Kasper EK Baughman KL Echocardiographic findings in fulminant and acute myocarditis. J Am Coll Cardiol. (2000) 36(1):227–32. 10.1016/S0735-1097(00)00690-2 10898439

[16] Serri K Reant P Lafitte M Berhouet M Le Bouffos V Roudaut R Global and regional myocardial function quantification by two-dimensional strain. J Am Coll Cardiol. (2006) 47(6):1175–81. 10.1016/j.jacc.2005.10.061 16545649

[17] Karlsen S Dahlslett T Grenne B Sjøli B Smiseth O Edvardsen T Global longitudinal strain is a more reproducible measure of left ventricular function than ejection fraction regardless of echocardiographic training. Cardiovasc Ultrasound. (2019) 17:1–12. 10.1186/s12947-019-0168-9 31477137 PMC6720884

[18] Chinali M Franceschini A Ciancarella P Lisignoli V Curione D Ciliberti P Echocardiographic two-dimensional speckle tracking identifies acute regional myocardial edema and sub-acute fibrosis in pediatric focal myocarditis with normal ejection fraction: comparison with cardiac magnetic resonance. Sci Rep. (2020) 10(1):11321. 10.1038/s41598-020-68048-5 32647322 PMC7347592

[19] Bucius P Erley J Tanacli R Zieschang V Giusca S Korosoglou G Comparison of feature tracking, fast-SENC, and myocardial tagging for global and segmental left ventricular strain. ESC Heart Fail. (2020) 7(2):523–32. 10.1002/ehf2.12576 31800152 PMC7160507

[20] Giusca S Korosoglou G Zieschang V Stoiber L Schnackenburg B Stehning C Reproducibility study on myocardial strain assessment using fast-SENC cardiac magnetic resonance imaging. Sci Rep. (2018) 8(1):14100. 10.1038/s41598-018-32226-3 30237411 PMC6147889

[21] Piccinelli E Herberg J Kang H Fraisse A Krupickova S Altamar IB Segmental and global longitudinal strain differences between children with paediatric inflammatory multisystem syndrome temporally associated with SARS-CoV-2 pandemic and Kawasaki disease. Eur Heart J. (2021) 22(Suppl 1):jeaa356.186. 10.1093/ehjci/jeaa356.186

[22] Sirico D Costenaro P Di Chiara C Dona D Cozzani S Fumanelli J Left ventricle longitudinal strain alterations in asymptomatic or mildly symptomatic pediatric patients with recent SARS-CoV-2 infection. Eur Heart J. (2021) 22(Suppl 1):jeaa356.167. 10.1093/ehjci/jeaa356.167 PMC9383392 35134865

[23] Supeł K Wieczorkiewicz P Przybylak K Zielińska M. 2D strain analysis in myocarditis-can we be any closer to diagnose the acute phase of the disease? J Clin Med. (2023) 12(8):2777. 10.3390/jcm12082777 37109114 PMC10146770

[24] Pruitt CR Menon S Lal AK Eckhauser AW Ou Z Presson A Usefulness of left ventricular myocardial deformation in children hospitalized for acute myocarditis who develop arrhythmias. Am J Cardiol. (2021) 152:113–9. 10.1016/j.amjcard.2021.04.041 34148631 PMC10103582

[25] Gursu HA Cetin II Azak E Kibar AE Surucu M Orgun A The assessment of treatment outcomes in patients with acute viral myocarditis by speckle tracking and tissue Doppler methods. Echocardiography. (2019) 36(9):1666–74. 10.1111/echo.14449 31452268

